# Reduced Neutrophil Apoptosis in Diabetic Mice during Staphylococcal Infection Leads to Prolonged Tnfα Production and Reduced Neutrophil Clearance

**DOI:** 10.1371/journal.pone.0023633

**Published:** 2011-08-30

**Authors:** Frank Hanses, Sunny Park, Jeremy Rich, Jean C. Lee

**Affiliations:** Channing Laboratory, Department of Medicine, Brigham and Women's Hospital and Harvard Medical School, Boston, Massachusetts, United States of America; Duke University Medical Center, United States of America

## Abstract

Diabetes is a frequent underlying medical condition among individuals with *Staphylococcus aureus* infections, and diabetic patients often suffer from chronic inflammation and prolonged infections. Neutrophils are the most abundant inflammatory cells during the early stages of bacterial diseases, and previous studies have reported deficiencies in neutrophil function in diabetic hosts. We challenged age-matched hyperglycemic and normoglycemic NOD mice intraperitoneally with *S. aureus* and evaluated the fate of neutrophils recruited to the peritoneal cavity. Neutrophils were more abundant in the peritoneal fluids of infected diabetic mice by 48 h after bacterial inoculation, and they showed prolonged viability *ex vivo* compared to neutrophils from infected nondiabetic mice. These differences correlated with reduced apoptosis of neutrophils from diabetic mice and were dependent upon the presence of *S. aureus* and a functional neutrophil respiratory burst. Decreased apoptosis correlated with impaired clearance of neutrophils by macrophages both *in vitro* and *in vivo* and prolonged production of proinflammatory tumor necrosis factor alpha by neutrophils from diabetic mice. Our results suggest that defects in neutrophil apoptosis may contribute to the chronic inflammation and the inability to clear staphylococcal infections observed in diabetic patients.

## Introduction

Diabetes mellitus is a group of metabolic disorders characterized by hyperglycemia. Patients with type 1 diabetes fail to produce insulin, and patients with type 2 diabetes develop resistance to insulin. The incidence of diabetes mellitus continues to rise, especially in developed countries [1], [2]. While the prevalence of diabetes currently lies between 6% and 10%, it is estimated that individuals born in the United States in the year 2000 have a risk of 1 in 3 to develop diabetes during their lifetime [3]. Although type 1 and type 2 diabetes differ in their respective etiologies, both forms share complications such as vasculopathy, nephropathy, retinopathy and neuropathy. Additionally, diabetes is associated with deficiencies in wound healing, chronic inflammation and enhanced susceptibility to infection. Diabetic foot infections are a major complication of diabetes mellitus [4], and chronic leg ulcers are a frequent cause for hospitalizations and amputations among diabetic patients. *Staphylococcus aureus* is a bacterial pathogen frequently implicated in these chronic infections. *S. aureus* nasal carriage, a known risk factor for staphylococcal disease, is higher among diabetic patients than healthy individuals [5], [6]. Invasive staphylococcal infections (such as endocarditis or bacteremia) are more prevalent in diabetic than in nondiabetic patients and are associated with a poor outcome in patients with diabetes [7], [8]. In studies of diabetic rodents, chronic wounds were characterized by tissue persistence of inflammatory cells, such as neutrophils [9], [10], and prolonged expression of proinflammatory cytokines [11]. Whereas some authors report impaired bactericidal function and decreased phagocytic activity by neutrophils from diabetic patients [12], [13], others have failed to demonstrate significant differences in neutrophil function in diabetic versus control patients [14]. Some of these conflicting findings may be explained by heterogeneous patient populations. Animal models of diabetes offer the advantage of examining the function and fate of neutrophils in defined models of infections [15]–[17].

Neutrophils are short-lived but abundant leukocytes. They are rapidly recruited to the site of a bacterial infection and are generally considered to be part of the “first line of defense” of the host innate immune system. Because of their sheer numbers, as well as their toxic contents and elaboration of proinflammatory cytokines, neutrophil clearance is key to the resolution of the inflammatory response and hence tightly regulated [18], [19]. Neutrophil apoptosis (either spontaneous or pathogen induced) is crucial for neutrophil uptake and subsequent elimination by macrophages at the site of infection, leading to resolution of the inflammatory process [20]. To address whether dysregulated neutrophil apoptosis during *S. aureus* infection might contribute to the severity and chronicity of bacterial infections observed in diabetic patients, we utilized a mouse model of invasive *S. aureus* infection in a diabetic host.

## Methods

### Ethics Statement

Animal experiments were performed in accordance with the guidelines of the Harvard Medical School Standing Committee on Animals (Animal Welfare Assurance Number A3431-01) under approved protocol 03565. The Harvard Medical School animal management program is accredited by the American Association for Accreditation of Laboratory Animal Care and meets National Institutes of Health standards as set forth in “Guide for the Care and Use of Laboratory Animals” (DHSS Publication No. (NIH) 85-23 Revised 1985). The institution also accepts as mandatory the Public Health Service “Policy on Humane Care and Use of Laboratory Animals by Awardee Institutions” and NIH “Principles for the Utilization and Care of Vertebrate Animals Used in Testing, Research and Training.”

### Mouse model of *S. aureus* infection

NOD mice were derived by Makino et al. [15] by selective inbreeding of a single female glucosuric mouse from a substrain of ICR mice. Female NOD mice spontaneously develop type 1 diabetes between 15 and 30 weeks of age [16], and by 20 weeks of age, 70–80% of females become diabetic. We obtained NOD mice from The Jackson Laboratories (Bar Harbor, ME), and some of the animals were bred in our facility. The mice were housed in a modified barrier facility under viral antibody-free conditions and were fed an autoclaved diet. Blood glucose levels were tested with glucostrips (Bayer, Elkhart, IN), and blood ketone levels were tested with PrecisionXtra β-Ketone Test strips (Abbott Laboratories, Alameda, CA). Nondiabetic mice were normoglycemic with blood glucose levels below 125 mg/dl (7 mmol/l). Diabetic mice had negative or low blood acetone levels, normal body weight, and blood glycemia levels ≥450 mg/dl (25 mmol/l) at the time of bacterial challenge.

Female diabetic (age 13–25 weeks) mice and control age-matched nondiabetic female littermates were challenged intraperitoneally (IP) with 10^8^ CFU *S. aureus* strain PS80 (a capsular serotype 8 isolate) cultured overnight on Columbia salt agar, and the animals were euthanized thereafter at the indicated time points. The peritoneal cavity was lavaged with 5 ml PBS, the total inflammatory cells were harvested, and a differential cell count determined. The *S. aureus* CFU/ml peritoneal fluid was determined by quantitative plate counts.

### Neutrophil isolation and *ex vivo* incubation

Neutrophils were isolated from peritoneal exudates of *S. aureus*-infected diabetic and nondiabetic mice by density gradient centrifugation. Peritoneal lavage fluids were overlaid on a 62% Percoll gradient and centrifuged for 25 min at 1200 g at room temperature. Contaminating erythrocytes were removed by hypotonic lysis. After two additional washes in PBS, viable neutrophils were enumerated by trypan blue exclusion, suspended in Minimum Essential Medium (MEM) supplemented with 10% fetal calf serum (FCS) and 100 ug/ml gentamicin (Gm) to prevent extracellular bacterial growth and incubated *ex vivo*. Neutrophil viability and apoptosis were measured at time zero and after 6 or 12 h of incubation at 37°C.

Alternatively, neutrophils were recruited to the mouse peritoneal cavity by IP injection of 1 ml of a 9% casein solution as a sterile inflammatory stimulus 18 h and again 3 h before euthanasia. Where indicated, neutrophils were stimulated *ex vivo* with *S. aureus* (pre-opsonized for 20 min with normal mouse serum stored at −80°C to preserve complement activity) at a bacterium to cell ratio of 1∶1 or 5∶1. After an incubation time of 1 h at 37°C, the neutrophils were washed, resuspended in MEM/FCS with 100 µg/ml Gm and further incubated at 37°C for 6 or 18 h.

### Apoptosis assays

Neutrophil apoptosis was assessed by three methods. Phosphatidylserine exposure on the outer cell membrane was measured using an annexin binding assay (BD Biosciences, San Jose, CA) according to the manufacturer's instructions. In brief, 1.5×10^5^ neutrophils were washed, resuspended in 100 µl PBS and labeled with PE-labeled Ly6G antibody (eBioscience, San Diego, CA) for 15 min on ice. After washing away unbound antibodies, cells were resuspended in 100 µl binding buffer, 5 µl FITC labeled annexin V and 5 µl 7-amino-actinomycin D (AAD) to exclude dead cells. After incubation for 20 min in the dark, the percentage of annexin(+), AAD(−), Ly6G(+) cells was analyzed by flow cytometry (BD FACSCalibur®). Nuclear condensation was measured by suspending neutrophils in PBS and air drying them on a microscope slide. Cells were stained for 10 min with 10 µg/ml Hoechst 33342 (Invitrogen, Carlsbad, CA), mounted in PBS/glycerol, and at least 200 cells were counted using fluorescence microscopy. Cells with condensed and fragmented nuclei were scored as apoptotic. DNA strand breaks were assessed with a TUNEL staining procedure (Roche Applied Science, Indianapolis, IN). In brief, 2×10^5^ neutrophils were fixed in cold paraformaldehyde, washed in PBS and permeabilized overnight in cold 70% ethanol in PBS. The neutrophils were washed in TUNEL dilution buffer, labeled with TUNEL reaction mix according to the manufacturer's protocol, washed and incubated with PE-labeled Ly6G antibodies. The percentage of TUNEL(+), Ly6G(+) cells was assessed by flow cytometry.

### Intracellular tumor necrosis factor alpha (TNFα) staining

Neutrophils were isolated as described above from the peritoneal lavage fluids of diabetic or nondiabetic mice 18 h after IP challenge with *S. aureus*. The cells were washed, suspended in MEM/FCS + 100 µg/ml Gm, and incubated for 3 h with Brefeldin A (1 ng/ml; GolgiPlug®, BD Biosciences). Alternatively, the isolated neutrophils were incubated *ex vivo* for 7 h at 37°C before a 3-h Brefeldin A treatment (leading to total incubation time of 10 h). TNFα production was measured using an intracellular cytokine staining procedure. The neutrophils were washed, fixed in 4% paraformaldehyde for 20 min, pelleted and resuspended in PBS/FCS for overnight storage at 4°C. After an additional wash, the neutrophils were suspended in Wash/Perm buffer (BD Biosciences) and labeled with FITC-labeled Ly6G and PE-labeled mouse TNFα antibodies (both eBioscience). After two washes, the neutrophils were suspended in PBS, and the percentage of Ly6G(+) TNFα (+) cells was determined by flow cytometry.

### Phagocytosis of neutrophils by macrophages – *in vitro*

Neutrophils isolated from peritoneal exudates of *S. aureus*-infected mice were labeled with CellTracker® Green (Invitrogen) for 15 min, washed, resuspended in MEM containing 100 µg/ml Gm, and incubated for 8 h at 37°C to induce apoptosis. Macrophages from the mononuclear fraction of the same exudates were adhered to glass cover slips in tissue culture plates at a concentration of 3×10^5^ cells/well, and nonadherent cells were washed away after 90 min. After a total incubation time of 8 h, adherent macrophages were overlaid with 3×10^6^ labeled neutrophils, incubated for 2 h and then washed thoroughly with PBS. The remaining adherent cells were stained with CellMask® Orange (Invitrogen), washed and fixed in paraformaldehyde. Slides were mounted in antifade solution (50% glycerol in PBS with 2% n-propyl-gallate) and examined by fluorescence microscopy. The percentage of macrophages with internalized neutrophils was calculated from a minimum of 200 cells counted.

### Phagocytosis of neutrophils by macrophages – *in vivo*

Neutrophils purified from peritoneal exudates of *S. aureus*-infected diabetic or nondiabetic mice were labeled for 15 min with CellTracker® Green, washed, and suspended in PBS. The labeled neutrophils (2×10^6^ neutrophils per mouse) were injected IP into diabetic or nondiabetic mice infected with 10^8^ CFU *S. aureus* for 20 h. Peritoneal exudates were recovered from the recipient animals 3 h later, and the mononuclear cells were purified, washed, and labeled with PE-labeled F4/80 antibodies (eBioscience). The cells were fixed in paraformaldehyde, and the percentage of macrophage-associated (F4/80+) CellTracker Green labeled neutrophils was determined by flow cytometry.

### Statistical analysis

**Figure 1:**
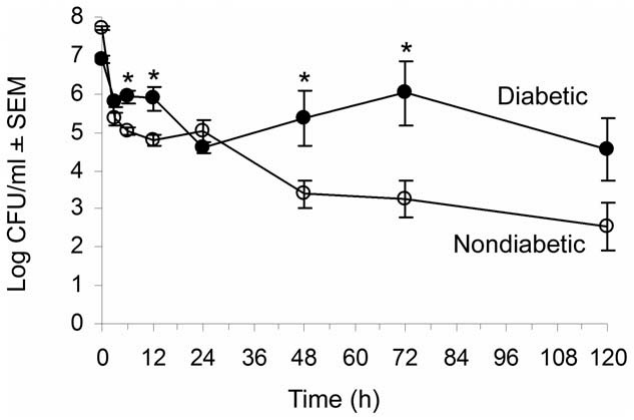
Diabetic mice show enhanced susceptibility to systemic *S. aureus* infection. Age-matched nondiabetic (∓) and diabetic (ℓ) mice were challenged IP with 10^8^ CFU *S. aureus* PS80. Results of quantitative cultures are shown as mean ± SEM CFU/ml peritoneal fluid recovered from 3–13 mice per group. * indicates *P*<0.05 by the unpaired Student t test.

**Figure 2:**
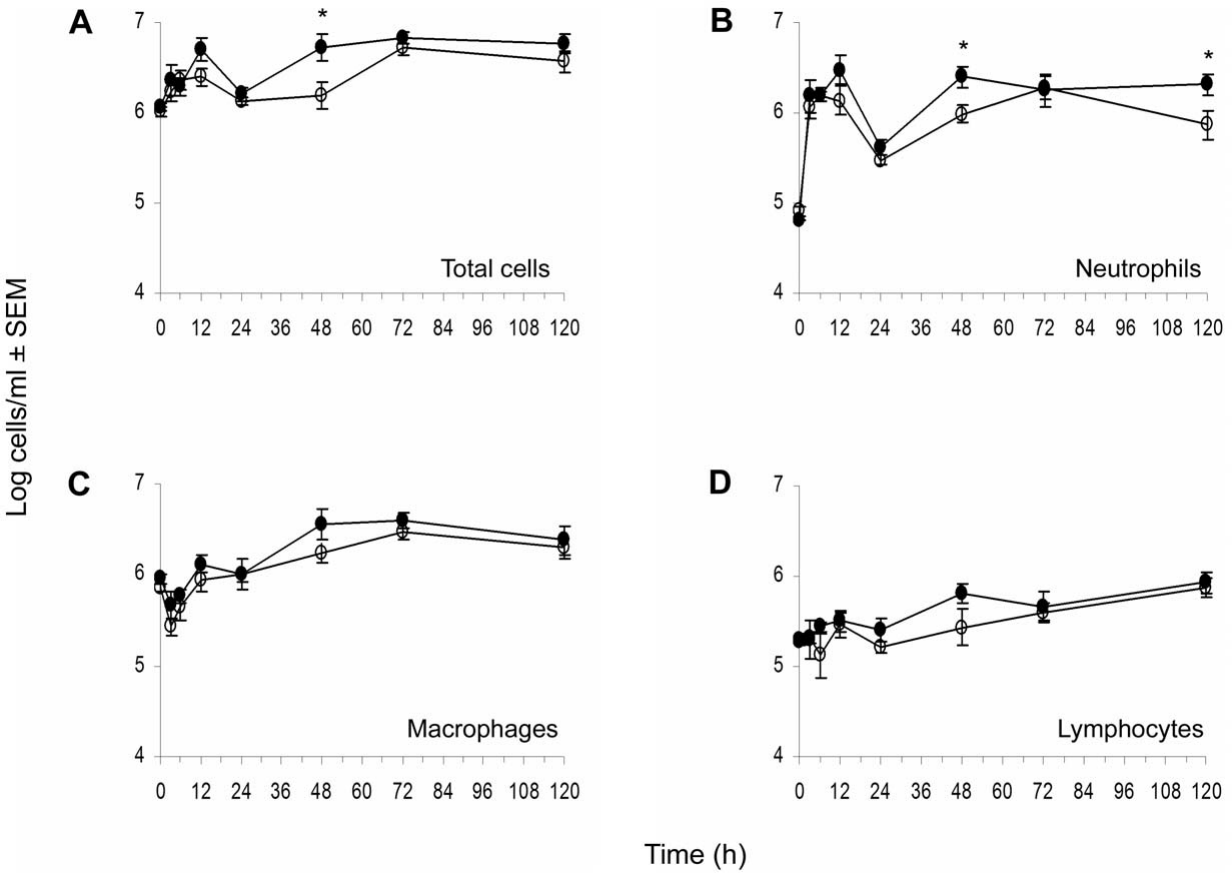
Cellular kinetics of peritoneal fluids recovered from *S. aureus* infected mice. At different time points, animals were euthanized, and peritoneal lavage fluids were collected. Total cell counts (A) were determined with a hemocytometer. For differential counts cytospin preparations of peritoneal cells were stained with Diff-Quik, and neutrophils (B), macrophages (C), and lymphocytes (D) were enumerated. ∓: nondiabetic mice, ℓ: diabetic mice. * indicates *P*<0.05 by the unpaired Student t test.

Results were expressed as means ± standard error of the mean (SEM), and Shapiro-Wilk tests were used to verify the assumption of a normally distributed data set. The data shown in Figs. 1 and 2 were analyzed by the unpaired Student t test. However, in all subsequent experiments each mouse was compared individually to an age-matched normoglycemic littermate since NOD mice developed diabetes between 13 and 25 weeks of age, . As a result, data sets comparing age-matched diabetic and nondiabetic mice were analyzed with a two-tailed paired Student t test. A *P*-value of <0.05 was considered to be statistically significant.

## Results

### Systemic model of *S. aureus* infection in nondiabetic and diabetic mice

Our previous studies revealed that a localized *S. aureus* PS80 infection of the hindpaw tissues was exacerbated in diabetic NOD mice compared to that of nondiabetic mice, and the diabetic animals were unable to clear the infection over a 10-day period. We observed delayed neutrophil recruitment to the site of infection in the diabetic mice, and this finding was consistent with chemokine levels in the infected hindpaw tissues of the diabetic mice that were significantly lower than those of age-matched nondiabetic littermates [17]. In this study age-matched nondiabetic or diabetic mice were challenged IP with 10^8^ CFU *S. aureus* PS80. All the mice developed a nonlethal acute peritonitis and were bacteremic. The diabetic mice showed impaired clearance of the systemic infection compared to the nondiabetic mice, who cleared the peritoneal infection within 5 days (Fig. 1).

To determine whether the delayed clearance of *S. aureus* from the peritoneal cavity and blood of diabetic mice was due to an impaired inflammatory response, we measured the numbers of total leukocytes recruited to the peritoneal cavity at various time points following *S. aureus* challenge. The total cell number in the peritoneal lavage fluid was increased from ∼10^6^ to 10^7^ cells/ml peritoneal fluid in both diabetic and nondiabetic mice after *S. aureus* inoculation, and the kinetics of cell migration were similar in both groups (Fig. 2A). Diabetic animals showed an increased bacterial burden at the early time points (<24 h post bacterial challenge), but no delay in neutrophil recruitment to the peritoneal cavity was observed (Fig. 2B). Diabetic mice showed significantly higher neutrophil counts in the peritoneal cavity 48 h and 120 h after challenge with *S. aureus* (Fig. 2). Overall, the percentage of each cell type - neutrophils, macrophages, and lymphocytes - recruited to the peritoneal cavity was similar in diabetic and nondiabetic animals.

### Enhanced viability of diabetic neutrophils

In an attempt to explain the failure of diabetic mice to clear the staphylococcal infection despite an apparently adequate inflammatory response, we assessed the bactericidal activity of neutrophils recruited to the peritoneal cavity following the administration of a sterile inflammatory stimulus (casein). No difference in killing capacity between the two mouse groups was apparent, since neutrophils from diabetic and nondiabetic mice killed 65–70% of opsonized *S. aureus* within 1 h *ex vivo*.

**Figure 3:**
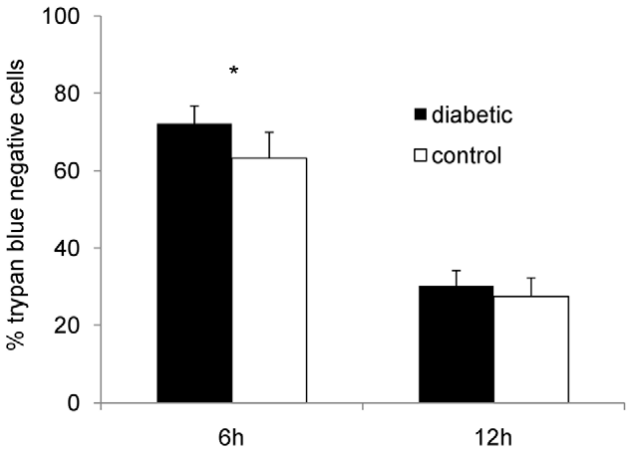
Viability of neutrophils isolated from the peritoneal cavities of diabetic and nondiabetic mice infected for 18 h with *S. aureus* PS80. Neutrophils were incubated *ex vivo* for 6 or 12 h, and the percentage of viable (trypan blue negative) neutrophils was measured. Mean percentages ± SEM from five independent experiments are depicted; * indicates *P*<0.05.

Because the neutrophils recruited to the peritoneal cavity of diabetic mice appeared functionally intact, we hypothesized that they might show impaired clearance from the infection site, and that this finding might correlate with the persistent inflammation observed in diabetic hosts. To test neutrophil viability without interference by variable bacterial numbers recovered *in vivo*, we isolated neutrophils from mouse peritoneal exudates 18 h after challenge with *S. aureus*, i.e. at a time point where bacterial loads in the peritoneal cavity were similar in diabetic and nondiabetic animals and similar numbers of neutrophils were recovered from the peritoneal lavage fluids. The neutrophils were incubated for either 6 or 12 h *ex vivo* at 37°C, and cell viability was assessed microscopically by trypan blue exclusion. Pair-wise comparisons showed that 72% of neutrophils from diabetic mice remained viable after 6 h compared with 63% of neutrophils from nondiabetic mice (Fig. 3; *P* = 0.039). This modest yet reproducible difference in viability was abrogated by 12 h, when only ∼30% of neutrophils from either group of mice remained viable.

### *S. aureus* induced apoptosis is reduced in neutrophils from diabetic mice

**Figure 4:**
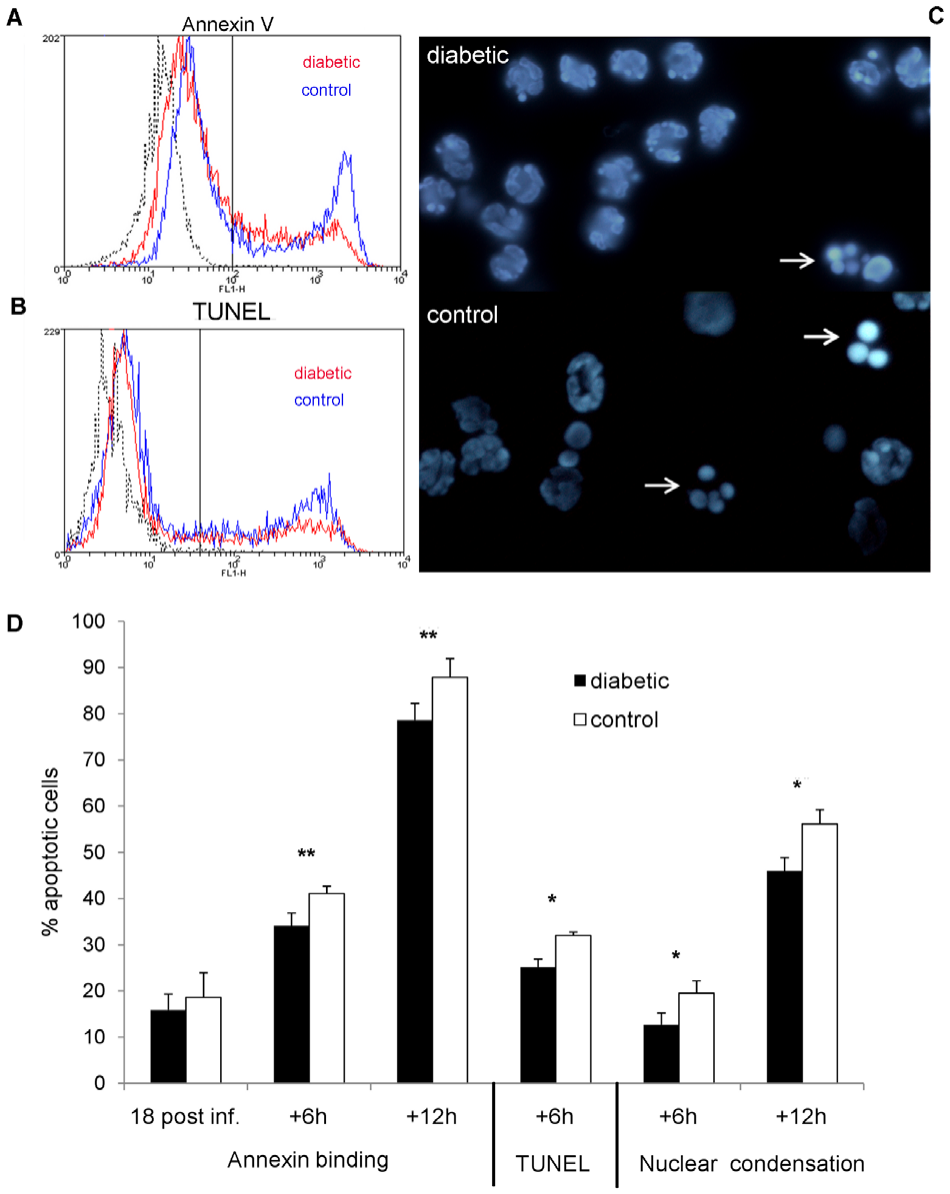
Neutrophil apoptosis—*in vivo* infection. Neutrophils were isolated from the peritoneal cavities of mice 18 h after IP challenge with 10^8^ CFU *S. aureus* and evaluated immediately or after incubation *ex vivo* for 6 or 12 h. A) Representative flow cytometric analysis of annexin V binding (A) or TUNEL staining (B) of Ly6G+ neutrophils from NOD mice that had been incubated *ex vivo* for 6 h. Black line represents unstained neutrophils. (C) Nuclear condensation observed in neutrophils from a diabetic and a nondiabetic mouse 6 h after isolation. White arrows indicate condensed and fragmented nuclei. (D) Quantitative comparison of annexin binding, TUNEL staining and nuclear condensation of neutrophils from age-matched diabetic and nondiabetic animals reveals that neutrophils from diabetic mice show reduced apoptosis compared to neutrophils from nondiabetic mice. Mean percent apoptotic cells ± SEM from 4 to 6 independent experiments are depicted. *P* values are calculated from pair-wise comparisons of age-matched mice; * indicates *P*<0.05, ** *P*<0.01.

Since neutrophil apoptosis is the main mode of cell death in mature neutrophils, we set out to test whether delayed apoptosis might contribute to the observed increase in the viability of neutrophils from diabetic mice. Direct examination of peritoneal exudate fluids revealed few apoptotic neutrophils, which may be due to efficient *in vivo* clearance by macrophages. Neutrophils, isolated 18 h after challenge with *S. aureus*, were subsequently incubated *ex vivo* for up to 18 h. Since some variability was observed among the experimental animals, we tested age-matched female diabetic and nondiabetic mice in pair-wise comparisons. Phosphatidylserine exposure (annexin V binding), DNA strand breaks (TUNEL staining) and nuclear condensation were measured as different hallmarks of cellular apoptosis. Neutrophils recovered from the peritoneal cavities of *S. aureus* infected diabetic mice (without additional incubation) showed similar annexin binding to neutrophils from control mice (15.9±3.4% versus 18.6±5.4%). However, upon further incubation *ex vivo*, neutrophils from diabetic mice bound significantly less annexin than neutrophils from nondiabetic mice at 6 h (*P* = 0.009) and 12 h (*P* = 0.0098) (Figs. 4A and 4D). Similarly, neutrophils from diabetic mice showed reduced TUNEL staining 6 h after isolation (*P* = 0.049; Figs. 4B and 4D) compared to neutrophils from diabetic mice. Nuclear condensation was likewise reduced at both 6 h (*P* = 0.032) and 12 h (*P* = 0.013) after isolation when neutrophils from diabetic and nondiabetic mice were compared (Figs. 4C and 4D). Together these results indicate that neutrophils from diabetic mice show a significantly reduced rate of apoptosis in response to *S. aureus* infection compared to neutrophils from age-matched nondiabetic mice (Fig. 4D).

**Figure 5:**
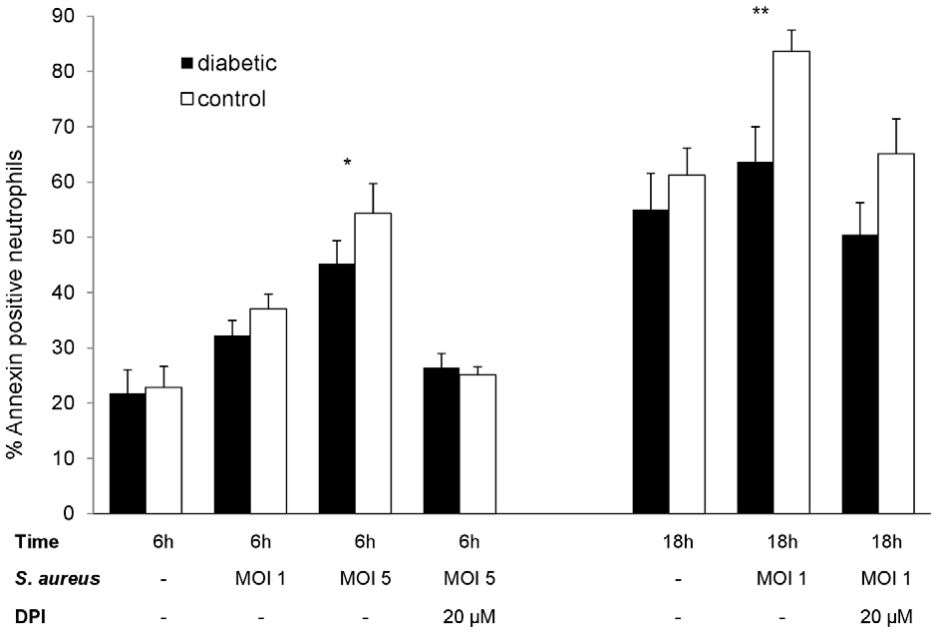
Neutrophil apoptosis—*ex vivo* infection. Apoptosis of neutrophils isolated from mouse peritoneal cavities after injection of casein as a sterile inflammatory stimulus. The cells were incubated *ex vivo* for 6 or 18 h with or without the addition of opsonized staphylococci. In the absence of *S. aureus*, no significant differences in annexin binding were observed. However, at a *S. aureus* MOI of 1, significantly fewer neutrophils from diabetic mice were apoptotic after 18 h; an MOI of 5 resulted in significant differences between the mouse groups by 6 h. No significant differences between neutrophils from diabetic and nondiabetic mice were found (at 6 h or 18 h) in the presence of 20 µM DPI. Mean percent apoptotic neutrophils ± SEM from 3 to 6 independent experiments are depicted. *P* values are calculated from pair-wise comparisons of age-matched mice, * indicates *P*<0.05, ** *P*<0.01.

To address whether the differences in neutrophil apoptosis were pathogen dependant or due to aberrant constitutive apoptosis, we evaluated the fate of neutrophils recruited to the peritoneal cavity following injection of casein. Neutrophils were isolated from murine peritoneal exudates and incubated *ex vivo* with or without pre-opsonized *S. aureus*. Spontaneous neutrophil apoptosis (as measured by percent annexin-positive cells) was similar for diabetic and nondiabetic mice at both 6 h (21.8±4.3% versus 22.9±3.8%) and 18 h (55.0±6.5% versus 61.2±5.0%) after isolation. When opsonized *S. aureus* cells were added to the murine neutrophil suspension at a multiplicity of infection (MOI) of 1, apoptosis of neutrophils from diabetic or nondiabetic mice was similar at 6 h (32.3±2.7% versus 37.0±2.7%). However, significantly (P<0.01) fewer apoptotic cells were found among neutrophils from diabetic animals than nondiabetic mice after 18 h (Fig. 5). Increasing the MOI to 5 led to noticeable differences as early as 6 h after exposure to *S. aureus* with significantly (*P* = 0.012) fewer annexin-positive cells in the diabetic compared to the nondiabetic sample. These results indicate that the decrease in apoptosis characteristic of neutrophils from diabetic mice was specific for neutrophils exposed to *S. aureus*.

We previously demonstrated that peripheral blood neutrophils from diabetic mice showed a diminished respiratory burst upon ingestion of *S. aureus* compared to neutrophils from nondiabetic mice [17]. A functional NADPH-oxidase is essential for the induction of apoptosis (as measured by phosphatidylserine exposure) upon ingestion of *S. aureus*, and a lack of NADPH-oxidase activity leads to elevated neutrophil numbers in the peritoneal cavities of mice challenged with heat-killed staphylococci [21]. To determine whether the reduced apoptosis observed in diabetic mice was due to a lower oxidative burst in neutrophils from diabetic animals, we incubated casein-elicited neutrophils from the peritoneal cavities of diabetic or nondiabetic mice with opsonized *S. aureus* in the presence or absence of diphenyleneiodonium (DPI), an inhibitor of the NADPH-dependant oxidase. As shown in Fig. 5, the differences in neutrophil apoptosis (measured by annexin binding) between diabetic and nondiabetic mice were reduced in the absence of a functional NADPH oxidase enzyme, suggesting that differences in the oxidative burst contribute to the reduced apoptosis of neutrophils from diabetic animals infected with *S. aureus*.

### Neutrophils from *S. aureus* infected diabetic mice fail to down-regulate cytokine production

**Figure 6:**
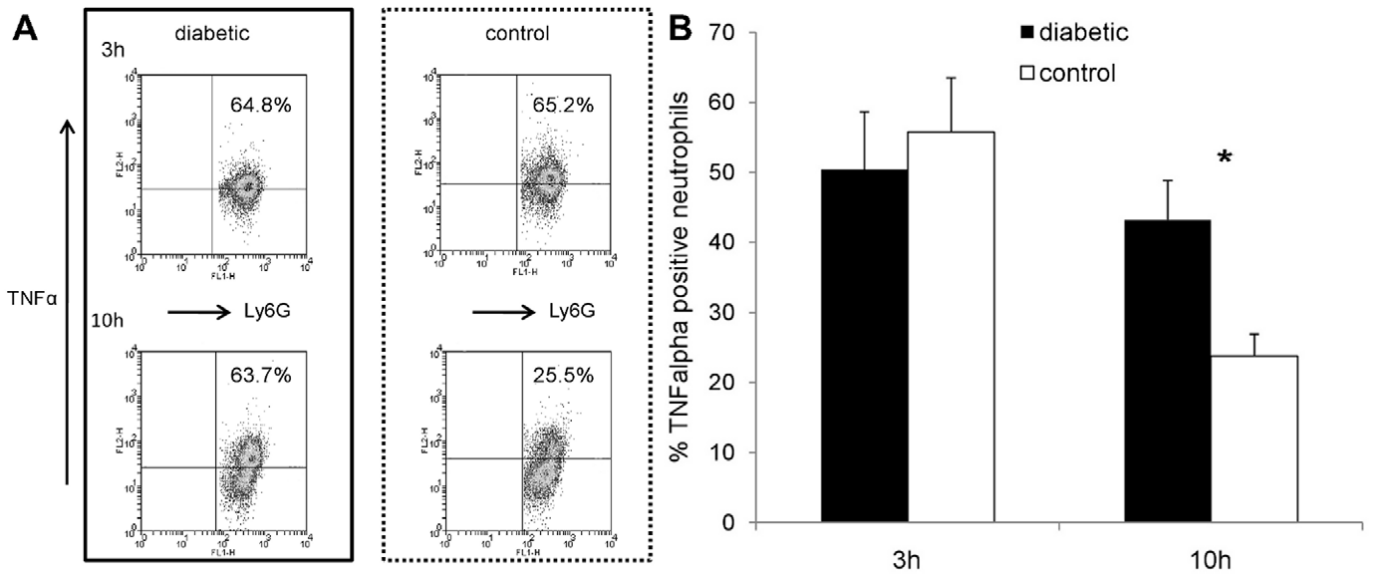
TNFα production by neutrophils isolated from the peritoneal cavities of mice 18 h after IP challenge with 10^8^ CFU *S. aureus* PS80. (A) Intracellular TNFα staining of neutrophils from a representative age-matched mouse pair 3 h (upper panels) or 10 h (lower panels) after neutrophil isolation. The upper right panels show the percentage of neutrophils that stained positive for TNFα production. (B) Whereas no significant differences were found between diabetic and nondiabetic mice after 3 h, diabetic animals had significantly more neutrophils positive for TNFα 10 h after neutrophil isolation. Data represent means ± SEM of data from six independent experiments. *P* values are calculated from pair-wise comparisons of age-matched mice; * indicates *P*<0.05.

Kobayashi et al. demonstrated that the expression of proinflammatory cytokines by human neutrophils was down-regulated during phagocytosis-induced apoptosis [22]. Failure to undergo apoptosis may thus lead to prolonged production of these inflammatory cytokines by neutrophils. To determine whether neutrophils from diabetic mice might show prolonged cytokine production due to delayed apoptosis, we measured intracellular TNFα (a representative proinflammatory cytokine) in neutrophils isolated from the peritoneal cavities of diabetic or nondiabetic mice 18 h after challenge with *S. aureus*. After *ex vivo* neutrophil aging, neutrophil expression of TNFα was detected using an intracellular cytokine staining procedure. Three hours after isolation, equivalent numbers of neutrophils from diabetic and nondiabetic mice stained positive for TNFα (Fig. 6). Whereas 55.8±7.7% of neutrophils from control animals were TNFα-positive after 3 h, only 23.8±3.2% stained positive after 10 h (*P* = .002). In contrast, no significant reduction in TNFα positive neutrophils from diabetic mice occurred over time (50.5±8.3% at 3 h versus 43.2±5.7% at 10 h, *P* = .59). Consequently, significantly (*P* = .036) more neutrophils from diabetic mice stained positive for TNFα than neutrophils from nondiabetic mice after 10 h (Fig. 6).

### Clearance of *S. aureus* stimulated neutrophils is reduced in diabetic mice

**Figure 7:**
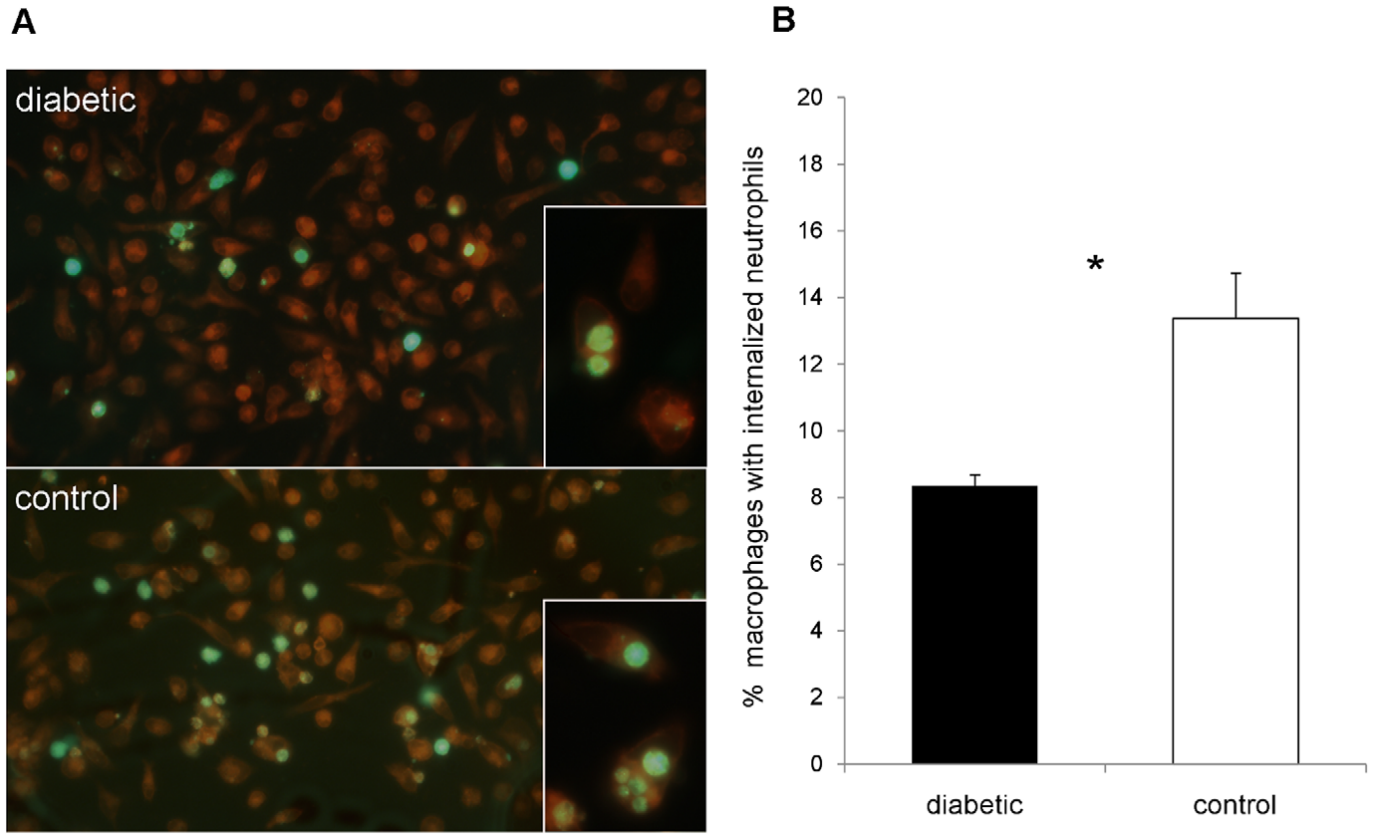
*In vitro* phagocytosis of apoptotic neutrophils by murine macrophages. Inflammatory cells were isolated from infected mouse peritoneal cavities 18 h after IP challenge with 10^8^ CFU PS80. The neutrophils were labeled with CellTracker Green, incubated in vitro for 8 h to induce apoptosis, and incubated with adherent autologous macrophages for 90 min to allow for uptake. (A) Representative image of CellMask Orange-stained macrophages with phagocytosed neutrophils from an age-matched pair of a diabetic (upper picture) and nondiabetic (lower picture) control mice. Phagocytosis of apoptotic neutrophils by macrophages from diabetic mice is less than that of macrophages from nondiabetic mice. Some neutrophils are still extracellular whereas others have been internalized. (B) Macrophages from diabetic mice ingested significantly fewer neutrophils than macrophages from nondiabetic control mice. Data are means ± SEM of six independent experiments; * indicates *P*<0.05.

Programmed cell death in neutrophils results in recognition, uptake and clearance by macrophages [20]. To determine whether neutrophil uptake and removal by macrophages differed between diabetic and nondiabetic animals, we designed in vitro and in vivo experiments. We isolated and labeled neutrophils recovered from the peritoneal cavities of *S. aureus*-infected diabetic and nondiabetic mice. For *in vitro* experiments, the neutrophils were aged for 8 h and then incubated for 2 h with adherent autologous macrophages. The macrophage cultures were washed, and cells with ingested fluorescent neutrophils were counted by microscopy. Samples from diabetic animals had significantly fewer macrophages with ingested neutrophils than samples from nondiabetic controls (8.4±0.3% versus 13.4±1.4%, *P* = 0.028; Fig. 7).

**Figure 8:**
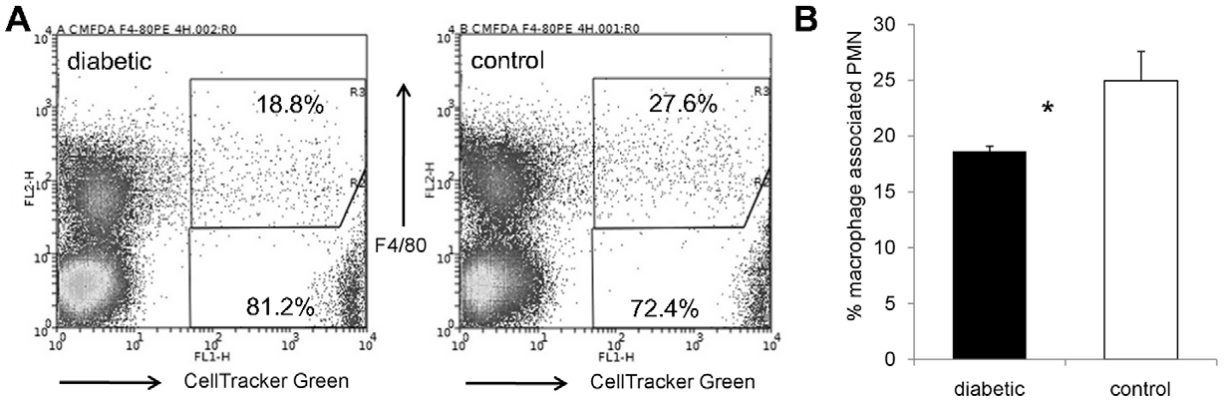
*In vivo* phagocytosis of apoptotic neutrophils by murine macrophages. Inflammatory cells were isolated from peritoneal exudates 18 h after IP challenge of NOD mice with 10^8^ CFU *S. aureus*. The neutrophils were labeled with CellTracker Green and injected measured IP into recipient diabetic or nondiabetic mice that were previously infected with *S. aureus*. Inflammatory cells from the recipient animals were collected 3 h later, macrophages were stained with F4/80-PE, and the percentage of macrophage-associated neutrophils was using flow cytometry. A) Flow cytometric analysis of inflammatory cells from a diabetic and a nondiabetic mouse. Macrophage-associated labeled neutrophils are in the upper right region; labeled neutrophils not associated with macrophages are depicted in the lower right region. Unlabeled cells from the recipient animals are depicted in the left regions. B) Significantly fewer neutrophils from diabetic mice were associated with macrophages compared to nondiabetic controls. The data represent the means ± SEM of results from six independent experiments; * indicates P<0.05.

Phagocytosis of apoptotic neutrophils by macrophages not only depends on the recognition of apoptotic neutrophils by macrophage receptors but also on several serum derived factors [23]. To validate our in vitro findings, we performed in vivo experiments whereby we injected labeled donor neutrophils (isolated from diabetic or nondiabetic mice 18 h after IP challenge with *S. aureus*) into corresponding (i.e. diabetic or nondiabetic) recipient animals 24 h after IP challenge with *S. aureus*. Peritoneal cavities were lavaged 3 h later, and the percentage of fluorescent neutrophils that colocalized with macrophages was measured by flow cytometry. Consistent with our *in vitro* data, diabetic animals showed fewer labeled neutrophils (20.8±2.0%) associated with macrophages than the nondiabetic control animals (30.5±3.6%; *P* = 0.026, Fig. 8).

## Discussion

The rising prevalence of both type 1 and type 2 diabetes worldwide [1], [2], [24] has led to an increasing incidence of diabetes-associated complications. While intensive research has been carried out on the epidemiology, pathogenesis and major complications of diabetes, the pathophysiology of infectious and inflammatory complications has received less attention. Ketoacidosis in patients with type 1 diabetes is frequently precipitated by infections [25], and diabetic patients in general are more susceptible to a variety of infections [26], [27], especially those caused by *S. aureus*. Diabetic patients have a higher rate of nasal colonization with *S. aureus* than healthy controls [28], [29], and *S. aureus* is frequently isolated from diabetic foot infections, which are a major cause for hospitalization of diabetic patients [30]. Diabetes is a risk factor for invasive *S. aureus* disease [8], including pneumonia [31]. Moreover, diabetes mellitus is the second most common underlying comorbidity in *S. aureus* bacteremia cohorts, and diabetes is associated with a poorer outcome both in bacteremia and endocarditis [32]–[34]. With regard to leukocyte function in diabetes mellitus, the available data are conflicting. Whereas some authors report differences in neutrophil metabolism [35], cytokine production [36], respiratory burst [17] or bacterial killing [37], [38], other studies failed to detect significant reductions in neutrophil function in diabetic hosts [39]. Delayed healing of chronic wounds in diabetic hosts has been linked to persistent inflammation reflected by high neutrophil and macrophage numbers and elevated levels of proinflammatory cytokines [9], [11].

NOD mice spontaneously develop autoimmune diabetes (resembling type 1 diabetes in humans) and present a valuable tool to study the pathogenesis of infectious diseases in a diabetic host. We utilized a mouse model of bacterial peritonitis to measure recruitment of neutrophils to the site of an invasive *S. aureus* infection and to monitor the fate of those neutrophils after bacterial uptake. Although the immediate influx of neutrophils to the peritoneal cavity was similar for nondiabetic and diabetic mice, more neutrophils persisted at the infection site 48 h after challenge in diabetic mice compared to nondiabetic littermates. This is in contrast to our findings in a localized *S. aureus* infection of the mouse hindpaw in which diabetic mice showed a delayed inflammatory response in the infected tissue compared to that of age-matched nondiabetic littermates [17].

In a systemic infection ongoing neutrophil recruitment due to a higher bacterial burden in the peritoneal cavity of diabetic mice cannot be reliably distinguished from an increase in neutrophil half-life. To overcome this dilemma, we collected and evaluated neutrophils at a time point when cell counts were not significantly different between diabetic and nondiabetic animals (i.e., 18 h). A limitation of our approach, however, is that monitoring neutrophil apoptosis requires an *ex vivo* experimental design to preclude loss of apoptotic cells through clearance by macrophages *in vivo*. There are likely differences between the conditions of the *ex vivo* incubations in comparison to the conditions within the peritoneal cavity, and such differences could alter the rate of neutrophil apoptosis.

Neutrophils isolated from the infected peritoneal cavities of diabetic mice showed prolonged viability *ex vivo* in trypan blue exclusion assays compared to that of nondiabetic mice. Neutrophils are abundant but short lived cells, and constitutive apoptosis is essential for regulating neutrophil homeostasis [18]. Whereas constitutive apoptosis can be delayed through extracellular proinflammatory agents such as Toll-like receptor agonists [40], neutrophils undergo rapid apoptosis following phagocytosis of bacteria [19]. This pathogen-induced cell death is dependent on the production of neutrophil NADPH-oxidase derived reactive oxygen species (ROS) without the need for traditional death receptors [41]. ROS generated via the NADPH oxidase are essential for phosphatidylserine exposure during cell death induced by phorbol myristate acetate, but not for neutrophils undergoing spontaneous apoptosis [42]. Likewise, *S. aureus* induced neutrophil apoptosis is dose- and time-dependant, mediated by ROS and greatly increased in the presence of viable staphylococci [43], [44]. The fate of professional phagocytes is modulated depending on the nature of the bacterial pathogen and the host cell type involved. Whereas induction of apoptosis in longer lived professional phagocytes (such as macrophages) may present a way of undermining the host immune response, neutrophil apoptosis upon phagocytosis of bacteria seems to be essential for the resolution of infection [45].

*S. aureus*-infected diabetic mice showed a significantly lower rate of neutrophil apoptosis than infected nondiabetic littermates at several time points after isolation. Reduced apoptosis was not observed in neutrophils elicited by a sterile stimulus, but it was dependent on the presence of both bacteria and a functional NADPH oxidase, which is in line with studies by other authors reporting normal spontaneous apoptosis in neutrophils from diabetic patients [46]. As studies with *S. aureus* and neutrophils from mice with chronic granulomatous disease [21] or mice lacking beta-integrins [41] have shown, a NADPH-oxidase deficiency leads to reduced neutrophil apoptosis and elevated neutrophil numbers in inflammatory exudates, consistent with impaired recognition and clearance. However, our study demonstrates that a different disease entity (diabetes) leads to reduced neutrophil clearance resulting from a moderately reduced ROS production [17] instead of a complete absence of NADPH oxidase activity. Neutrophils from the blood of hyperglycemic NOD mice exhibit a diminished respiratory burst in response to *S. aureus* [17], and this may contribute to reduced pathogen-induced apoptosis in diabetic mice, resulting in higher neutrophil counts in peritoneal exudate fluids. Clinical studies have found a decreased rate of neutrophil apoptosis (eventually promoting tissue injury) in a patient group with major diabetic complications [47]. Previous studies have focused on the inability of diabetic patients to delay constitutive apoptosis of neutrophils upon stimulation with LPS [36], [48].

During the induction of apoptosis, neutrophils undergo significant alterations in gene expression profiles [22], including several genes encoding inflammatory cytokines. Consistent with reduced apoptosis, we showed that neutrophils from *S. aureus* infected diabetic mice failed to down regulate the production of TNFα over time. The failure to limit the production of mediators potentially causing tissue injury might contribute to the extensive tissue damage observed during infections in diabetic patients.

The clearance of neutrophils and their toxic contents, which could be deleterious for the surrounding tissue, is an important prerequisite for the resolution of infection. Neutrophil uptake by macrophages and their subsequent degradation is an important means of limiting tissue injury associated with inflammation [20]. Phosphatidylserine is externalized to the outer membrane layer during apoptosis and participates in neutrophil clearance by macrophages [49], a process that does not seem to be inhibited by high concentration of glucose [50]. Without apoptosis, neutrophil effector function is preserved and internalization by macrophages is prevented [51]. The ingestion of apoptotic neutrophils not only leads to the removal of cytotoxic content, but it also modulates the host immune response [52]. Previous studies have shown that after neutrophils engulf *S. aureus*, phosphatidylserine exposure occurs, resulting in neutrophil uptake by macrophages [21]. We compared these processes in diabetic and nondiabetic mice both *ex vivo* and *in vivo*. Under both conditions, we observed fewer *S. aureus*-stimulated neutrophils within macrophages from diabetic mice than nondiabetic mice. Macrophages from 5–6 wk old normoglycemic NOD mice showed reduced phagocytosis of apoptotic thymocytes when compared to macrophages from BALB/c mice [53]. However, our comparisons were made between infected age-matched diabetic and nondiabetic mice of the same genetic background, and thus the lower rate of neutrophil clearance by macrophages could be correlated with the diabetic state of the host. Gresham et al. showed that *S. aureus* may survive inside mouse neutrophils *in vivo*, and that infected neutrophils were sufficient to establish an infection when transferred to naïve animals [54]. Hence, diminished neutrophil apoptosis after uptake of *S. aureus* and subsequent impaired clearance by macrophages may contribute to the persistent nature of staphylococcal infections observed in diabetic patients. Our studies suggest that reduced neutrophil apoptosis contributes to the chronic inflammation, extensive tissue injury and persistent staphylococcal infections observed in diabetic hosts.
